# Examining the Role of Effective Population Size on Mitochondrial and Multilocus Divergence Time Discordance in a Songbird

**DOI:** 10.1371/journal.pone.0055161

**Published:** 2013-02-15

**Authors:** Brian Tilston Smith, John Klicka

**Affiliations:** 1 School of Life Sciences, University of Nevada, Las Vegas, Las Vegas, Nevada, United States of America; 2 Marjorie Barrick Museum of Natural History, University of Nevada, Las Vegas, Las Vegas, Nevada, United States of America; Institute of Evolutionary Biology (CSIC-UPF), Spain

## Abstract

Estimates of speciation times are subject to a number of potential errors. One source of bias is that effective population size (N_e_) has been shown to influence substitution rates. This issue is of particular interest for phylogeographic studies because population sizes can vary dramatically among genetically structured populations across species’ ranges. In this study, we used multilocus data to examine temporal phylogeographic patterns in a widespread North American songbird, the Northern Cardinal (*Cardinalis cardinalis*). Species tree estimation indicated that the phylogeographic structure of *C. cardinalis* was comprised of four well-supported mainland lineages with large population sizes (large N_e_) and two island lineages comprised of much smaller populations (small N_e_). We inferred speciation times from mtDNA and multilocus data and found there was discordance between events that represented island-mainland divergences, whereas both estimates were similar for divergences among mainland lineages. We performed coalescent simulations and found that the difference in speciation times could be attributed to stochasticity for a recently diverged island lineage. However, the magnitude of the change between speciation times estimated from mtDNA and multilocus data of an older island lineage was substantially greater than predicted by coalescent simulations. For this divergence, we found the discordance in time estimates was due to a substantial increase in the mtDNA substitution rate in the small island population. These findings indicate that in phylogeographic studies the relative tempo of evolution between mtDNA and nuclear DNA can become highly discordant in small populations.

## Introduction

Reconstructing the phylogeographic history of species is reliant on having accurate estimates of speciation times. However, there are a number of factors that may bias speciation time estimates, such as gene flow [1], [2] gene tree discordance [3], ancestral effective population size [4], time-dependent substitution rates [5], behaviour [6], and improper molecular clock calibrations [7]. An issue that has received less attention is the influence of effective population size (N_e_) on substitution rates. Studies have shown substitution rates can vary among closely related species or even within species that have different N_e_ [8]. This issue is of particular interest for phylogeographic studies because population sizes in species can vary dramatically across their ranges.

Effective population size (N_e_) will dictate the relative strength of selection or drift on the fixation of mutations in a population [9]. The nearly neutral model of evolution predicts that slightly deleterious mutations will become fixed at a higher rate in small populations [10]. In larger populations, these slightly deleterious mutations are presumed to be removed by purifying selection. The interplay between selection and drift can be seen in the accumulation of different types of mutations in protein coding regions of DNA. These DNA substitutions result in either a change in the amino acid (nonsynonymous change) that is subject to selection or a redundant change in the codon sequence (synonymous change) that presumably does not affect fitness. One approach that attempts to explain the variation observed in amino acid changes is the estimation of the nonsynonymous/synonymous substitution rate ratio (dN/dS) between lineages with different sized N_e_.

Analyses of the relationship between N_e_ and the dN/dS ratio have not shown a consistent pattern [11], [12]. A range of studies have compared substitution rates across mammalian orders [13], endosymbiotic and free-living bacteria and fungi [14], island-mainland birds [15], and insects [16], [17]. A general consensus from these studies is that small populations have elevated substitution rates and high dN/dS ratios. However, evolutionary rate patterns may be biased when data are not phylogenetically independent and comparisons are made between evolutionary distant taxa with differing ecologies, selection regimes, and metabolic rates [12]. A recent study on island/mainland birds that presumably addressed many of these limitations found the opposite pattern of increased substitution rates in large mainland populations [12]. These contradictory results are surprising and may indicate that there are additional yet unrecognized factors that may bias findings.

The impact of gene-species tree discordance and ancestral N_e_ are often not accounted for in studies that assess substitution rate heterogeneity among small and large populations. Phylogenetic trees inferred from a single gene or concatenated multilocus data may be inadequate for inferring relationships among species [18]. Even when multiple genes are used, the data are often combined into a single sequence fragment and patterns of substitution rate variation occurring across genes and lineages may not be directly apparent. Additionally, the stochastic sorting of ancestral polymorphisms can give the appearance that substitution rates have increased [19] or indicate that divergence times occurred much earlier than inferred [4].The development of coalescent methods that estimate species trees from multilocus data [20], [21] can be used to infer more accurate estimates of evolutionary rates among sister lineages, and can be incorporated into a comparative framework to test whether an apparent rate increase is due to an actual increased substitution rate or if instead, the rate only appears to be accelerated due to randomness associated with the coalescent process.

In this study, we constructed a multilocus species tree of the widespread North American songbird, the Northern Cardinal (*Cardinalis cardinalis*). The phylogeographic structure of this species consists of multiple mainland and island lineages [22], which can be used as a case study for investigating the impact of N_e_ on speciation time estimates. We used a multilocus coalescent approach to evaluate speciation time patterns among island and mainland lineages and to test the hypothesis that population size impacts the actual rate of DNA substitutions. Further, we evaluated whether substitution rate shifts across lineages were present in both the mitochondrial and nuclear markers. To complete these objectives, we performed a series of tests that began by constructing gene and species trees and then comparing speciation times estimated from mtDNA and multilocus data, and finally, assessing possible causes of discordance between speciation times estimated from both data sets.

## Methods

### Ethics Statement

This study was approved by the Institutional Animal Care and Use Committee (IACUC) and in incompliance with IACUC guidelines.

### Taxa and Molecular Markers

**Figure 1:**
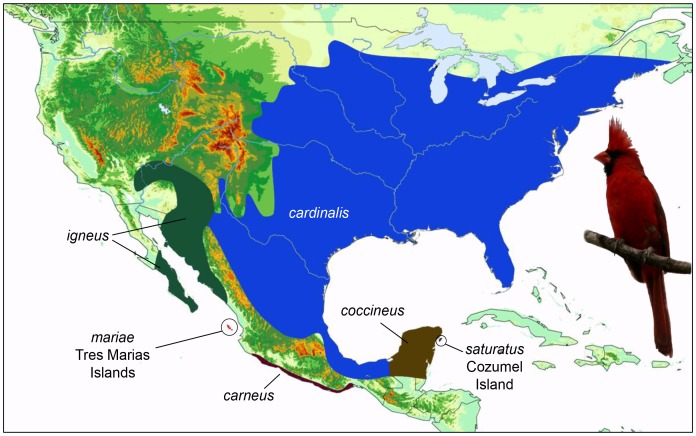
Range map of the *Cardinalis cardinalis* lineages. Approximate distributions of mainland and island *C. cardinalis* lineages. An image of a male *C. cardinalis igneus* is shown.

We selected 4–6 individuals from each of the six *C. cardinalis* lineages [22] and included six other closely related species (*C. sinuatus*, *C. phoeniceus*, *Rhodothraupis celaeno*, *Periporphyrus eyrthromelas*, *Caryothraustes poliogaster*, and *C. canadensis* and *Molothrus ater* as an outgroup ([23]; Data S1). *Cardinalis cardinalis* is widely distributed throughout the eastern United States and Mexico. The distribution of the six lineages are: *cardinalis* – eastern half of North America to eastern Mexico; *carneus* – Pacific coast of Mexico from Colima to Oaxaca; *igneus* – Sonoran desert to Baja peninsula; *mariae* – Tres Marias Islands; *coccineus* – Yucatan peninsula; and *saturatus* – Cozumel Island (Fig. 1). We extracted total genomic DNA from tissues using the DNeasy tissue extraction kit (Qiagen, Valenica, CA) and we amplified nine nuclear markers via polymerase chain reaction (PCR) in 12.5 µl reactions using the following protocol: denaturation at 94°C for 10 min, 40 cycles of 94°C for 30 s, 54°C for 45 s, and 72°C for 2 min, followed by 10 min elongation at 72°C and 4°C soak. We adjusted annealing temperatures for each intron (see Data S1 for complete gene names): ACA, ACO1, βact3, FGB-I5, MYC 60°C; EEF2 58°C; HMGN2 56°C; ODC 65°C; RHO-I1 61°C. We incorporated the mtDNA gene ND2 into our dataset from a previously published study [22] and additionally we sequenced cytochrome *b* for a subset of *C. cardinalis* individuals in order to estimate relative substitution rates between cytochrome *b* and ND2. PCR products were sent to the High-Throughput Genomics Unit (University of Washington) for all subsequent steps. PCR products were purified using ExoSAP-IT (USB Corporation, Cambridge, MA), run through cycle-sequencing reactions and final products were sequenced using BigDye (Applied Biosystems, Foster City, CA) on a high-throughput capillary sequencer. We aligned chromatograms in Sequencher 4.9 (GeneCodes Corporation, Ann Arbor, MI).

To resolve introns that had insertion/deletion events between homologous nuclear alleles, we used the program Indelligent [24]. To phase heterozygous sites in the nuclear introns, we performed three separate runs for each marker in the program PHASE v. 1.2 [25]. Haplotypes that had posterior probabilities of <0.90 were not included in subsequent analyses. We tested for recombination using the six different recombination tests in the program RDP3 [26].

### Gene Trees, Species Tree, and Divergence Times

We determined the best-fit sequence model for each gene based on the Akiake Information Criterion (AIC) scores from Mr.Modeltest v. 2.3 [27]. To construct gene trees, we used MrBayes v. 3.1.2 [28] and ran this analysis for 10,000,000 generations with four runs. To determine whether loci were evolving at a constant rate, we performed likelihood-ratio tests in PAUP* 4.0b10 [29] using both the Jukes Cantor and best-fit substitution models. Because there are no informative fossils for our system, we used the 2% avian clock as the basis for estimating divergence times. Additionally, we did not use a geologic calibration because of the potential bias that may be caused by the disparity between island and mainland substitution rates. We calculated mean relative substitution rates for each marker [30] by estimating uncorrected genetic distances from a common outgroup (*C. sinuatus*) in PAUP* 4.0b10 [29] and scaled each rate relative to the cytochrome *b* gene rate 2% per million years [31]. In order to compare branch lengths among cardinal phylogroups, we extracted the branch length from each cardinal phylogroup to *C. sinuatus* in each of the MrBayes gene trees using the program Mesquite [32].

We constructed a species tree using the program *BEAST [21].The program requires *a priori* designation of species or populations, so we used the six *C. cardinalis* lineages that are supported by both mtDNA [22] and morphology [33]. We performed several analyses to look at the impact of partitioning the mtDNA by codon position, the effects of strict and relaxed clocks, and estimated divergence times using all the markers (nuclear DNA and mtDNA), only nuclear DNA, and only mtDNA. For the nuclear markers, we used published avian intron rates [34]. We specified lognormal distributions for the relaxed uncorrelated rate priors: ND2 (mean = 0.0123 substitutions/site/million year; SD = 0.45), the autosomal markers (mean = 0.0018 substitutions/site/million year; SD = 0.45), and the sex-linked marker (mean = 0.00195 substitutions/site/million year; SD = 0.45). *BEAST was run for 200,000,000 generations sampling every 1,000 generations. All analyses implemented a Yule process on the species tree prior and lognormal distributions on sequence model prior distributions. For gene and species trees, we assessed MCMC convergence and determined burn-ins by examining ESS values, and likelihood plots in the program Tracer v. 1.5 [35], and we also assessed convergence of gene trees by inspecting the standard deviation of the split frequencies in MrBayes.

We performed an additional divergence time analysis using the program MCMCcoal [36]. The method implements the Jukes Cantor sequence model and assumes no gene flow among species. MCMCcoal uses a gamma (α,β) distribution to specify the prior distribution for the parameter θ, the population mutation rate parameter (θ = 4N_e_μ for a diploid locus, where μ is per nucleotide site per generation) and τ, the species divergence time parameter (τ = Tμ; T = species divergence time in millions of years). The shape parameter (α) and scale parameter (β) has a mean α/β and variance s^2^ = α/β^2^. All priors were scaled to the ND2 substitution rate and we set θ (1, 100) to have a mean N_e_ of 150,000 with a generation time of one year. We used the date of the re-emergence of Cozumel Island, 121,000 years ago, [37] to set the prior distribution (1, 670) for the divergence of the Cozumel Island lineage from the mainland Yucatan peninsula lineage. For the other nodes we did not have strong prior knowledge so we set the τ prior (1, 40) to have a mean divergence in the Early Pleistocene and an interval that covers much of the Pleistocene and Late Pliocene, a period often linked to the onset of intraspecific diversification in birds [38]. We performed several preliminary runs with other reasonable τ prior distributions that suggested the priors were not strongly influencing our results, and ran the final analyses for 100,000 generations with a burn-in of 10,000 generations. We measured the difference between speciation times estimated from mtDNA and multilocus using the equation
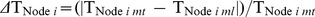
where node *i* represents a node in the *C. cardinalis* species tree and *mt* is the speciation estimated from mtDNA only and *ml* is the speciation time estimated from multilocus data.

We additionally used the program IMa that allows gene-flow with divergence [39] to estimate speciation times between the island-mainland lineages (*mariae*-*igneus* and *saturatus*-*coccineus*). To optimize parameter estimation, we evaluated a range of prior values for population sizes, gene flows and divergence time to obtain reasonable priors. For our final analyses, we ran the programs for 2,000,000 generations and with a burnin of 500,000 using four chains and a geometric heating scheme (−g1 = 0.90 and –g2 = 0.78). This analysis was constrained to the nuclear DNA because there was no haplotype sharing in the mtDNA. We assumed a generation time of one year and used the substitution rates specified in the *BEAST analyses. The posterior estimates of speciation time for *saturatus*-*coccineus* did not stabilize; therefore we constrained migration to 0 for the runs with this pair.

### Coalescent Simulations

To examine the impact of coalescent variance on speciation time estimated from mtDNA for the two island-mainland divergences, we performed coalescent simulations using the program MCcoal in the MCMCcoal package [36]. For each island-mainland divergence we specified a two population species tree and simulated a gene tree and nucleotide sequence data. To specify the τ and θ parameters for our simulations we used the mean multilocus MCMCcoal output of nodes D and E, and adjusted for the difference in N_e_ between mtDNA and nuclear DNA by dividing by 4. We performed 100 simulations of a single 1041 bp locus in MCcoal using the Jukes-Cantor sequence model and the same number of individuals as the empirical data. We then used the simulated nucleotide sequence data to estimate speciation times in the MCMCcoal [36] with the same prior settings from our analyses of the empirical data, except that we ran the analyses for 50,000 generations. We repeated this procedure for each simulated nucleotide sequence data set, recorded the mean τ estimate for each simulation, and built a null distribution of expected speciation times for the *saturatus*-*coccineus* and *mariae*-*igneus* divergences. We assessed whether observed mean speciation times estimated from mtDNA were significantly different from our simulated distributions by calculating the proportion of simulated values equal or higher to our observed data [40]. The goal of the test was to determine whether the difference in speciation times estimated from mtDNA and multilocus data for the two island-mainland divergences could be explained by coalescent stochasticity.

### Substitution Rates

We used the program Crann 1.04 [41] to estimate the rate of nonsynonymous (dN) and synonymous (dS) amino acid substitutions for the *C. cardinalis* lineages. To evaluate the relative tempo of evolution between lineages with small N_e_ versus lineages with large N_e_, we compared the dN/dS ratio for each island lineage to its sister mainland lineage. We compared the ratios between island-mainland sister lineages ([dN_island_/dS_island_]/[dN_mainland_/dS_mainland_], with values above 1 indicating faster island evolution. Additionally, we estimated if any of the codons were under positive selection using the HyPhy program [42] within the MEGA 5 package [43]. The program estimated dN and dS for each codon in ND2 and tested for positive selection using the test statistic dN-dS. Positive dN-dS test statistics indicate an overabundance of nonsynonymous substitutions.

## Results

### Genes and Trees

Locus characteristics are listed in Table 1. After we removed unphased haplotypes and omitted samples that would not amplify the following samples sizes for *C. cardinalis* were included: ACA 57/74; ACO1 72/74; βact3 54/74; EEF2 44/74; FGB-I5 68/74; HMGN2 47/74; MYC69/74; ODC 68/74; RHO 70/74 (number included/total number). DNA sequences are available on GenBank (KC313394–KC313887). There was considerable variation in the number of variable and parsimony informative sites across loci. Mean relative substitution rates ranged from 3.97×10^−9^ to 6.4×10^−10^ (substitution/sites/year) for the autosomal markers and 1.02×10^−9^ for the z-linked marker ACO1. These rates are of similar magnitude to the autosomal (1.8×10^−9^) and z-chromosome (1.95×10^−9^) rates estimated from the turkey and chicken genomes [31]. The mean rate of ND2 was set to 1.23×10^−8^ [22]. The six independent recombination tests detected recombination at only one sample in *C. sinuatus* for the HMG locus. Individual nuclear gene trees showed little topological resolution, whereas the ND2 gene tree was well resolved (Fig. S1). The clock test using the Jukes Cantor (used in MCMCcoal) and best-fit sequence model (used in *BEAST) allowed us to use the appropriate model for each of our divergence time analysis. Most loci did not evolve in clock-like fashion when the best-fit sequence models were used, but only HMG and ACA failed the clock-like substitution rate test using the Jukes Cantor model (Table S1). Due to the rejection of the molecular clock, we ran *BEAST with relaxed and strict clocks.

**Table 1:** Locus information.

locus	chrom.	base pairs	sub. model	var. sites	pars. inform. sites	GC content (%)	mean sub. rate
**ACA** a	1	1160	HKY+I+G	166	104	54.0	1.29×10^−9^
**ACO1** a	Z	947	GTR+G	124	84	38.6	1.02×10^−9^
**βact3** b	10	414	HKY+G	70	49	49.0	2.18×10^−9^
**EEF2** a	28	729	HKY+I+G	101	63	54.1	2.05×10^−9^
**FGB-I5** a	4	559	HKY+G	56	35	38.3	1.02×10^−9^
**HMGN2** a	23	788	GTR+I+G	83	43	43.6	3.97×10^−9^
**MYC** c,d	1	724	HKY+G	59	36	46.0	7.68×10^−10^
**ND2** e	mito	1041	GTR+I+G	385	349	45.7	1.23×10^−8^
**ODC** f	3	676	HKY+G	78	40	38.7	6.4×10^−10^
**RHO-I1** a	12	280	HKY+I	34	15	59.0	1.54×10^−9^

Locus characteristics including best-fit sequence model, chromosome, and substitution rate. Substitution rates were estimated relative the rate of the mtDNA rate. Chromosome (chrom.); substitution model (sub. model); variable sites (var. sites); parsimony informative sites (pars. inform. sites); mean substitution rate - substitution/sites/year (mean sub. rate).

a [45].

b [50].

c [49].

d [52].

e [22].

f [51].

**Figure 2:**
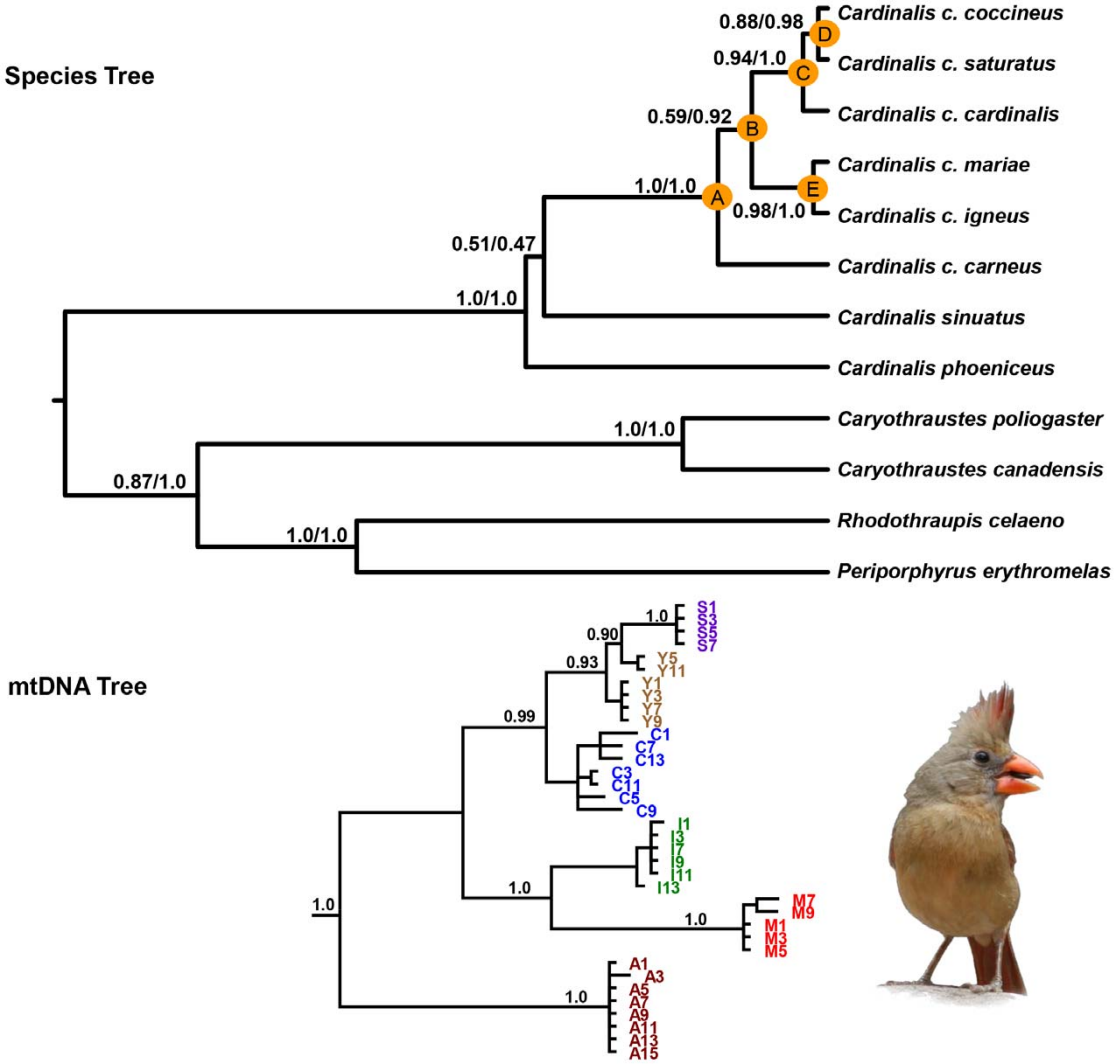
Multilocus species tree and mtDNA tree for *Cardinalis cardinalis* and allies. Species tree estimated from *BEAST using nuclear only data set, and nuclear and mtDNA combined data set (top). Posterior probabilities (PP) are reported for each node (left PP – nuclear DNA only species tree; right PP –mtDNA and nuclear DNA species tree) and the nuclear and mtDNA combined data set tree is shown. Letters on the species tree correspond to divergences among *C. cardinalis* lineages: A) *carneus* – all other lineages; B) *igneus*/*mariae* – *cardinalis*/*coccineus*/*saturatus*; C) *cardinalis* – *coccineus*/*saturatus*; D) *coccineus* – *saturatus*; E) i*gneus* – *mariae*. Nodes D and E refer to the island-mainland divergences. Shown on the bottom is the Bayesian mtDNA (ND2) tree of *C. cardinalis* lineages with PP plotted on nodes: S – *saturatus* (purple); Y – *coccineus* (brown); C – *cardinalis* (blue); I - *igneus* (green); M - *mariae* (red); A – *carneus* (maroon). An image of a female *C. cardinalis igneus* is shown.

The *BEAST analyses produced the same topology as the mtDNA gene tree (Fig. 2). Species tree analysis run without the mtDNA produced the same topology with support values similar (results not shown) to those obtained for the mtDNA and nuclear DNA combined species tree. The species tree indicated that the genus *Cardinalis* is sister to a fully resolved clade containing the genera *Rhodothraupis*, *Periporphyrus*, and *Caryothraustes*. There was weak support (Posterior Probability (PP) = 0.47) for relationships among the three *Cardinalis* species (*C. cardinalis*, *C. phoeniceus*, and *C. sinuatus*) in the species tree, a result consistent with the mtDNA tree. Within *C. cardinalis*, *C. c. carneus* is sister (Node A; PP = 1.0) to all other *C. cardinalis* lineages. The next break in the tree (Node B) separates the western clade (*C. c. igneus* and *C. c. mariae*) from the eastern clade (*C. c. cardinalis*, *C. c. coccineus*, and *C. c. saturatus*) and support for this node differed considerably across analyses (PP = 0.92). In the eastern clade, there is high support (Node C; PP = 1.0) for *C. c. cardinalis* (*cardinalis*) being sister to *C. c. coccineus*/*C. c. saturatus*, and high support (Node D; PP = 0.98) for *C. c. coccineus* and *C. c. saturatus* being sister. Within the western clade there is strong support for the *C. c. igneus* and *C. c. mariae* sister relationship (Node E; PP = 1.0).

### Effective Population Sizes

MCMCcoal and IMa analyses found that the two island populations had smaller N_e_ than mainland populations. Mean estimates of N_e_ from mtDNA were similar across the six extant lineages likely due to the low information content in the mtDNA (Table 2). However, mean estimates of N_e_ from multilocus data indicated that island lineages had lower N_e_ than mainland taxa (Table 2). The mean N_e_ estimate from multilocus data for *coccineus* was larger than estimates for *saturatus*, but it had a 95% credible interval that was overlapping with the N_e_ estimate for *saturatus* (Table 2). IMa N_e_ estimates indicated island lineages had smaller Ne (*mariae*: N_e_ 0.05 million, 0.01–0.09 95% HPD; *saturatus*: N_e_ 0.20 million, 0.05–0.42 95% HPD) than their mainland sisters, respectively (*igneus*: N_e_ 0.29 million, 0.07–0.74 95% HPD; *coccineus*: N_e_ 0.57 million 0.13–1.06 95% HPD).

**Table 2:** Effective population sizes of extant and ancestral lineages.

lineage	mean (mtDNA)	95% CI	mean (multilocus)	95% CI
N_e_ C	0.675	(0.321–1.236)	0.850	(0.514–1.319)
N_e_ A	0.104	(0.016–0.299)	0.583	(0.423–0.778)
N_e_ I	0.185	(0.047–0.465)	0.593	(0.291–1.043)
N_e_ M	0.199	(0.051–0.516)	0.197	(0.096–0.346)
N_e_ S	0.049	(0.002–0.217)	0.132	(0.024–0.343)
N_e_ Y	0.142	(0.026–0.413)	0.311	(0.069–0.713)
N_e_ SY	0.217	(0.010–0.699)	0.963	(0.437–1.598)
N_e_ SYC	0.189	(0.004–0.671)	1.274	(0.748–1.917)
N_e_ MI	0.207	(0.004–0.758)	0.752	(0.461–1.130)
N_e_ SYCMI	0.199	(0.006–0.720)	0.907	(0.321–1.638)
N_e_ SYCMIA	0.193	(0.004–0.701)	0.305	(0.057–0.685)

Effective population sizes for mtDNA and multilocus data. N_e_ units are in millions of individuals. *Cardinalis cardinalis* extant and ancestral lineages: *cardinalis* (C); *carneus* (A); *igneus* (I); *mariae* (M); *saturatus* (S); *coccineus* (Y). Ancestral lineages are represented by the common ancestor of daughter lineages.

### Comparative Speciation Times

**Figure 3:**
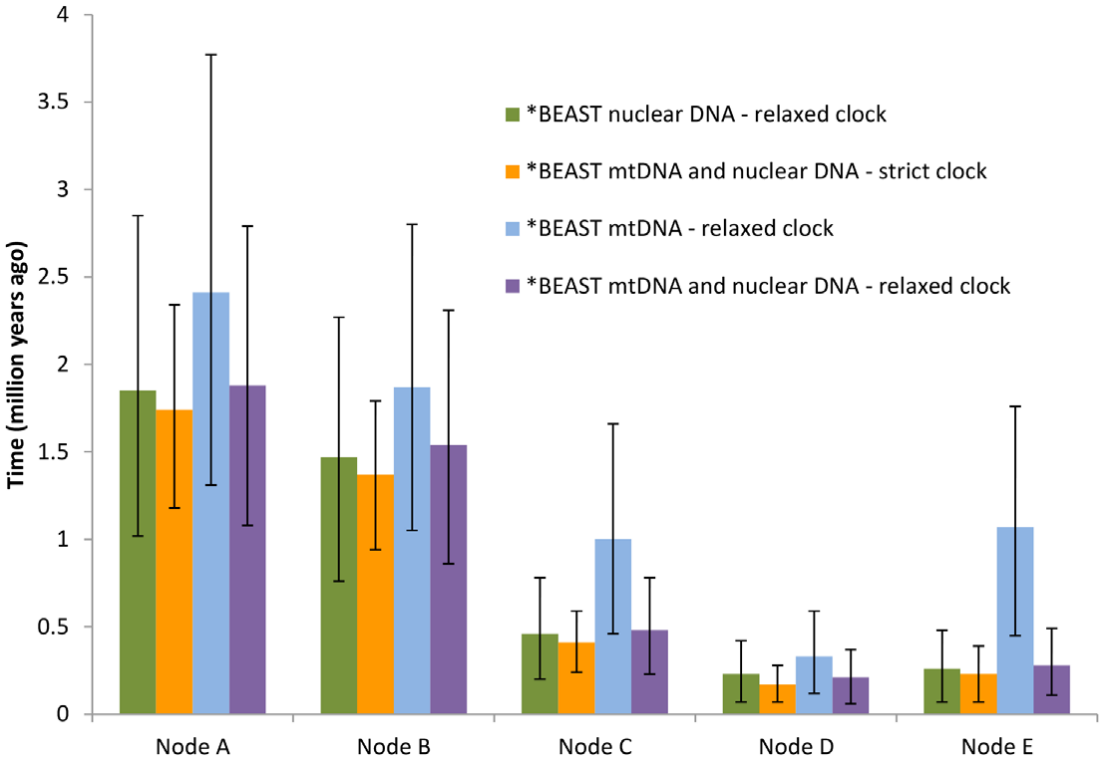
*BEAST speciation times estimated from the *Cardinalis cardinalis* species tree. Speciation time estimates using differing parameters and data sets: green - speciation times estimated from nuclear DNA using relaxed clocks, orange - speciation times estimated from mtDNA and nuclear DNA using strict clocks, light blue - speciation times estimated from mtDNA only using a relaxed clock, and purple - speciation times estimated from mtDNA and nuclear DNA using relaxed clocks. The nodes are from the *C. cardinalis* species tree: Node A) *carneus* – all other lineages; Node B) *igneus*/*mariae* – *cardinalis*/*coccineus*/*saturatus*; Node C) *cardinalis* – *coccineus*/*saturatus*; Node D) *saturatus*–*coccineus*; Node E) *mariae–igneus*.

**Figure 4:**
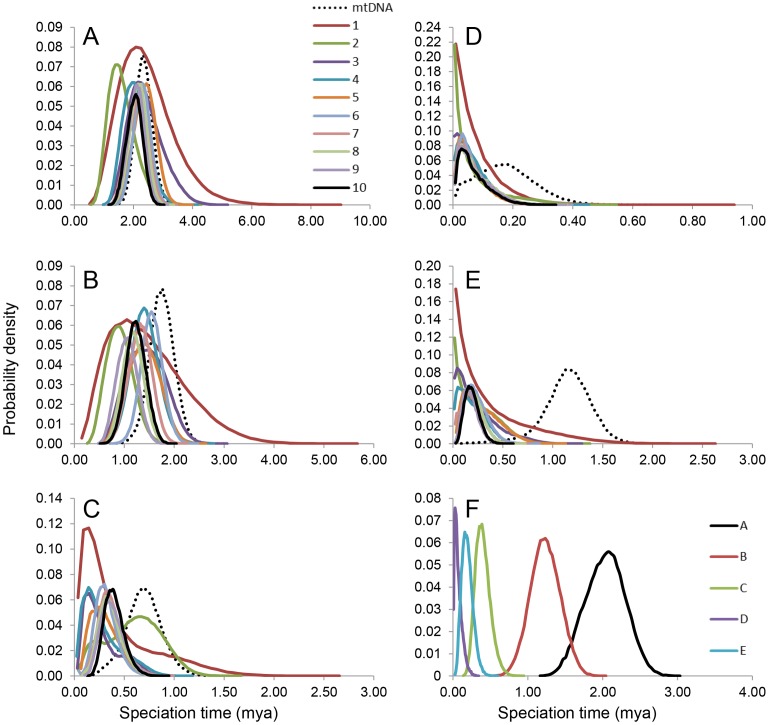
Density plots of posterior distributions of speciation times from MCMCcoal. Each graph represents a species tree node and the colors correspond to the number of loci used to estimate speciation time, million years ago (mya). A) *carneus* – all other lineages; B) *igneus*/*mariae* – *cardinalis*/*coccineus*/*saturatus*; C) *cardinalis* – *coccineus*/*saturatus*; D) *saturatus* – *coccineus*; E) *mariae – igneus*; F) speciation time estimates for each node. Nodes D and E refer to the island-mainland divergences.

Speciation time estimates from mtDNA were generally older than estimates from multilocus data (Fig. 3 & 4; Table S2). Partitioning ND2 by codon positions had a minimal impact on speciation time estimation (Table S2; 2A–5B). The MCMCcoal (Table S2 1B) and *BEAST speciation times estimates yielded similar results except for the *saturatus*/*coccineus* node (Table S2). The removal of ND2 from speciation time estimates did not impact the results (Table S2; 4A & 4B). The prior distribution (1, 670) based on Cozumel Island’s geology produced speciation time estimates similar (Table S2; 1A and B D*) to those obtained in an analysis using an Early Pleistocene prior (1, 40). We further evaluated whether our *BEAST speciation time estimates were biased by removing haplotypes that had low posterior probabilities for heterozygous sites by running a *BEAST analysis with just homozygous alleles and also a run that included phased haplotypes with low posterior probabilities. We recovered similar speciation times (results not shown) to the results presented in Fig. 3 and the speciation time at node D was younger than the estimate for the node using only mtDNA.

IMa time estimates for the divergence of the two island lineages were similar to our other estimates. The IMa time estimate with gene-flow of the *mariae-igneus* divergence was 0.20 mya (0.04–0.40 95% HPD). However, the posterior distributions of migration parameters for *mariae-igneus* did not stabilize. The IMa time estimate with no gene flow for *saturatus*-*coccineus* was 0.13 mya (0.04–0.22 95% HPD).

### Coalescent Variance

**Figure 5:**
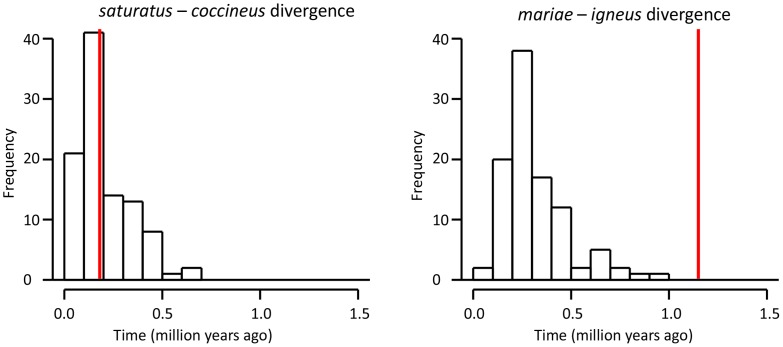
Coalescent simulations for island-mainland divergences. Simulated distributions of speciation times for island-mainland divergences: A) *saturatus* – *coccineus* divergence, and B) *mariae* – *igneus* divergence. Observed speciation times estimated from mtDNA for each divergence are plotted as vertical red lines.

The difference between mean speciation time estimates from mtDNA and multilocus data were variable across nodes (Fig. 4; ΔT_Node A_ = 13%; ΔT_Node B_ = 30%; ΔT_Node C_ = 43%; ΔT_Node D_ = 60%; ΔT_Node E_ = 83%). The two island lineages had the lowest multilocus N_e_ values (Table 2) and the largest ΔT. The mean speciation time from mtDNA for Node D, the divergence of *saturatus* and *coccineus*, was not significantly different from the simulated distribution (P = 0.98; Fig. 5), however the mean speciation time from mtDNA for Node E, the divergence of *mariae* and *igneus*, was significantly different from the simulated distribution (P = 0.00; Fig. 5).

### Substitution Rates

**Figure 6:**
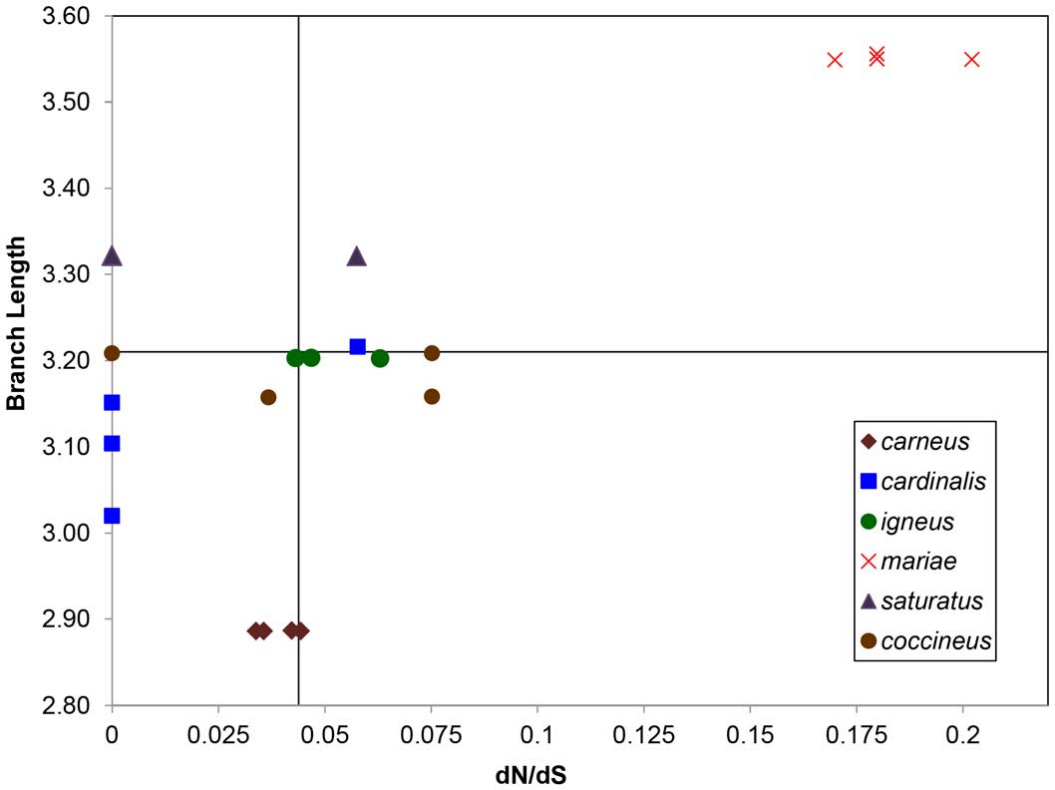
Mitochondrial DNA nonsynonymous/synonymous substitution rate ratio plotted against lineage branch length. Results for multiple haplotypes per lineage are shown. The median branch length is represented by the horizontal line and median dN/dS is represented by the vertical line. Branch lengths were estimated from the distance to common outgroup, *Cardinalis sinuatus*.

Mitochondrial DNA branch lengths for island taxa were longer than the mean branch length for mainland taxa, but this pattern was not observed across the nuclear markers (Fig. S2). Disparate branch lengths appeared to be randomly distributed across nuclear gene trees (Fig. S2). The median dN/dS ratio was 0.04 with the island lineage *mariae* having the highest ratio of 0.18 (Fig. 6; Table S3). The ratio of island/mainland dN/dS for *mariae*/*igneus* was 3.39, whereas it was only 0.31 for *coccineus*/*saturatus*. We found five fixed amino acid changes in the *mariae* lineage. The selection test found no evidence of positive selection (Data S2). Of the 15 codon positions with positive dN-dS test statistics no values were significantly different (α<0.05) from the null hypothesis of neutral evolution (Data S2).

## Discussion

We inferred a species tree for a widespread songbird, the Northern cardinal (*Cardinalis cardinalis*), and examined speciation time patterns and potential causes of discordance between estimates from mtDNA and multilocus data. Overall, we found that our results were highly consistent across methodologies, and robust to model parameterization, relaxing the molecular clock, and loci used. The most striking result from our analyses was the discordance between speciation time estimates from mtDNA and multilocus data of the divergence of island populations from their mainland source populations. In both cases, speciation time estimates from mtDNA of island colonization times were older than multilocus estimates. Moreover, this discordance in speciation times was less pronounced between diverging mainland populations. Despite the incongruence between speciation time estimates among data sets for some divergences in the *C. cardinalis* species tree, the topology of the species tree and mtDNA gene tree were congruent.

We assessed several possible causes of incongruence between speciation time estimates. The disparity in time estimates was likely not attributable to nuclear gene flow because the isolation-with-migration (IMa) analysis of the divergence of *mariae*-*igneus* found similar results to the *BEAST and MCMCcoal analyses that assumed no gene flow. Additionally, there was no evidence that the mtDNA was under positive selection. As for the role of coalescent stochasticity, we found mixed results between the two island colonisations. For the Cozumel Island divergence (*saturatus-coccineus*), the incongruence between mtDNA and multilocus time estimates was consistent with coalescent stochasticity. In contrast, the coalescent simulations suggested that the discordance between speciation time estimates for the colonization of the Tres Marias Islands (*mariae*-*igneus* divergence) were much greater than predicted. We found that the discordance was due to the mtDNA in the *mariae* lineage having a high dN/dS ratio (Fig. 5) and an approximate five-fold substitution rate increase. Overall, these results indicate that the relative tempo of evolution between different loci can become highly discordant in small populations.

The lack of a general pattern between the two island lineages is unclear at this time, but there are several apparent differences between the island lineages. The difference in N_e_ between sister lineages are greater between the *mariae/igneus* pair than the *saturatus/coccineus* pair. Moreover, speciation times indicate that the *mariae* lineage has likely been evolving independently from its mainland sister longer than the *saturatus* lineage has from its mainland sister. This may reflect a lag between island colonization and when substitution rates begin increasing, indicating that there has not been enough time for the substitution rate to increase in the *saturatus* lineage. Currently, comparative analyses with co-distributed species are limited because of a lack of multilocus studies on the taxa distributed on the Tres Marias Islands and Cozumel Island. One available comparison is *Forpus cyanopgyius,* a species that occurs in mainland Mexico and the Tres Marias Islands. Multilocus speciation times of a Tres Marias Islands’ colonization by *F. cyanopygius* were similar to the estimates for the *mariae* lineage, but there was no evidence of accelerated evolution in the mtDNA [44].

### Discordant Substitution Rates between Genomes

The approximate four-fold difference in N_e_ between mtDNA and nuclear DNA is a likely explanation for why the nuclear DNA does not show elevated substitution rates in the *mariae* lineage, however, there are differences between the loci that need to be acknowledged. The mtDNA gene we used was a coding gene, whereas, the majority of the polymorphisms in the nuclear markers were in non-coding introns. Although, it would be desirable to assess how protein coding nuclear genes evolve on islands, most published exons [45] do not exhibit enough polymorphisms to be included in island-mainland studies that generally focus on relatively shallow time scales. For example, we screened a relatively long exon (RAG1; 2,884 bp) and there was only one fixed difference between two species (*C. cardinalis* and *C. sinuatus*) in this study. Despite our inability to assess patterns of evolution in coding portions of the nuclear genome, rates of substitution were elevated in both nonsynonymous and synonymous sites in the mtDNA (Table S3). Elevated substitution rates in nonsynonymous and synonymous sites in the *mariae* lineage provides additional evidence that the disparity in rates of evolution between mtDNA and nuclear DNA are likely not attributed to the mtDNA being a protein coding gene.

Another major distinction between the mtDNA and nuclear DNA is that the mtDNA has a faster mutation rate. The slower mutation rate of the nuclear DNA may contribute to our findings, because there are fewer mutations in the nuclear DNA that can be fixed. However, the influence of mutation rate appears to have less of an impact than the difference in N_e_ between the two genomes. Coalescent theory predicts that the coalescent time of a neutral locus is determined by N_e_ and this value is independent from the marker’s mutation rate (see discussion in [46]). The influence of larger N_e_ on the nuclear DNA can be observed in the gene trees; of all ten loci the mtDNA is the only gene tree that exhibits a monophyletic *mariae* lineage (Fig. S1). Given enough time, it is unclear if nuclear substitution rates may begin to increase in small populations. Even if substitution rate changes are time-dependent in nuclear DNA, the magnitude of the rate shift will likely be less than in mtDNA given the four-fold difference in N_e_.

### Old Taxa on Young Islands

Our results suggest that single locus speciation times estimated from lineages with small N_e_ may be subject to greater error than lineages with large N_e_. This issue is particularly problematic when attempting to correlate island colonization with island geology. We found that the divergences of the two cardinal island lineages (*saturatus* and *mariae*, respectively) pre-dated the Late Pleistocene emergence of Cozumel Island [37] and the Tres Marias Islands [47] when only mtDNA was used. Estimating the age of island founder events is often challenging because the timing and pattern of colonization may be unclear and inferred founder events are frequently “older” than the island [for a review see [48]). Our results for the divergence of the Tres Marias Islands lineage indicated that the problem of “old taxa on young islands” may be due to undetected elevated substitution rates and highlight the need for examining the magnitude of nonsynonymous DNA changes when speciation times are estimated for small populations.

### Conclusion

Understanding the tempo of DNA substitutions across species and genomes is important for reconstructing the divergence of lineages across the landscape. A growing body of literature has shown that effective population size may influence a species substitution rate, but it is unclear whether accelerated substitution rates are attributed to populations being large or small. Additionally, it is not apparent if substitution rate shifts occur across multiple portions of a species genome. In this study, we used a multilocus coalescent framework to analyse both the tempo of evolution between large and small populations, and between mitochondrial and nuclear DNA. We found that small populations are subject to both, stochastic effects that give the appearance of a rate increase and actual elevated substitution rates. Importantly, our data suggests that small population size can affect mitochondrial and nuclear DNA differently. The fast evolving mtDNA makes it susceptible to accelerated evolution in small populations, whereas, slower-evolving nuclear DNA appears to be buffered against these effects. These findings indicate that the relative tempo of evolution of mtDNA compared to nuclear DNA can become highly discordant and bias interpretations in phylogeographic studies.

## Supporting Information

Figure S1 **Consensus gene tree generated in MrBayes.** Posterior probabilities are shown are nodes. A) ACA; B) ACO1; C) βact3; D) EEF2; E) FGB-I5; F) HMGN2; G) MYC; H) ND2; I) ODC; J) RHO-I1.
(PDF)

Figure S2 ***Cardinalis cardinalis*** **mean branch lengths.** Branch lengths were extracted from gene trees constructed using MrBayes. The branch length was the distance from each lineage to a common outgroup, *Cardinalis sinuatus*. Mean branch lengths were generated by averaging across all the individuals within a lineage.
(PDF)

Table S1 **Molecular clock likelihood ratio tests.** Shown are locus name, sequence model, and likelihood scores for clock constrained and unconstrained for each gene tree. Gene trees that are inconsistent with the molecular clock are shown in bold.
(PDF)

Table S2 **Speciation time estimated from mtDNA and multilocus data.**
(PDF)

Table S3 **Substitution rates and branch lengths extracted from the mtDNA gene tree.**
(PDF)

Data S1 **Appendix containing taxon list, specimen numbers, haplotype code, specimen localities, and information on genetic markers.**
(XLS)

Data S2 **Output from the positive selection test on mtDNA.**
(XLS)
