# Supplementary material for: Examining the Role of Effective Population Size on Mitochondrial and Multilocus Divergence Time Discordance in a Songbird
Source: PLoS One. 2013 Feb 15;8(2):e55161. doi: 10.1371/journal.pone.0055161 (PMC3574149; doi:10.1371/journal.pone.0055161)
Supplement: Figure S1 — Consensus gene tree generated in MrBayes. Posterior probabilities are shown are nodes. A) ACA; B) ACO1; C) βact3; D) EEF2; E) FGB-I5; F) HMGN2; G) MYC; H) ND2; I) ODC; J) RHO-I1. (PDF) [file pone.0055161.s001.pdf]

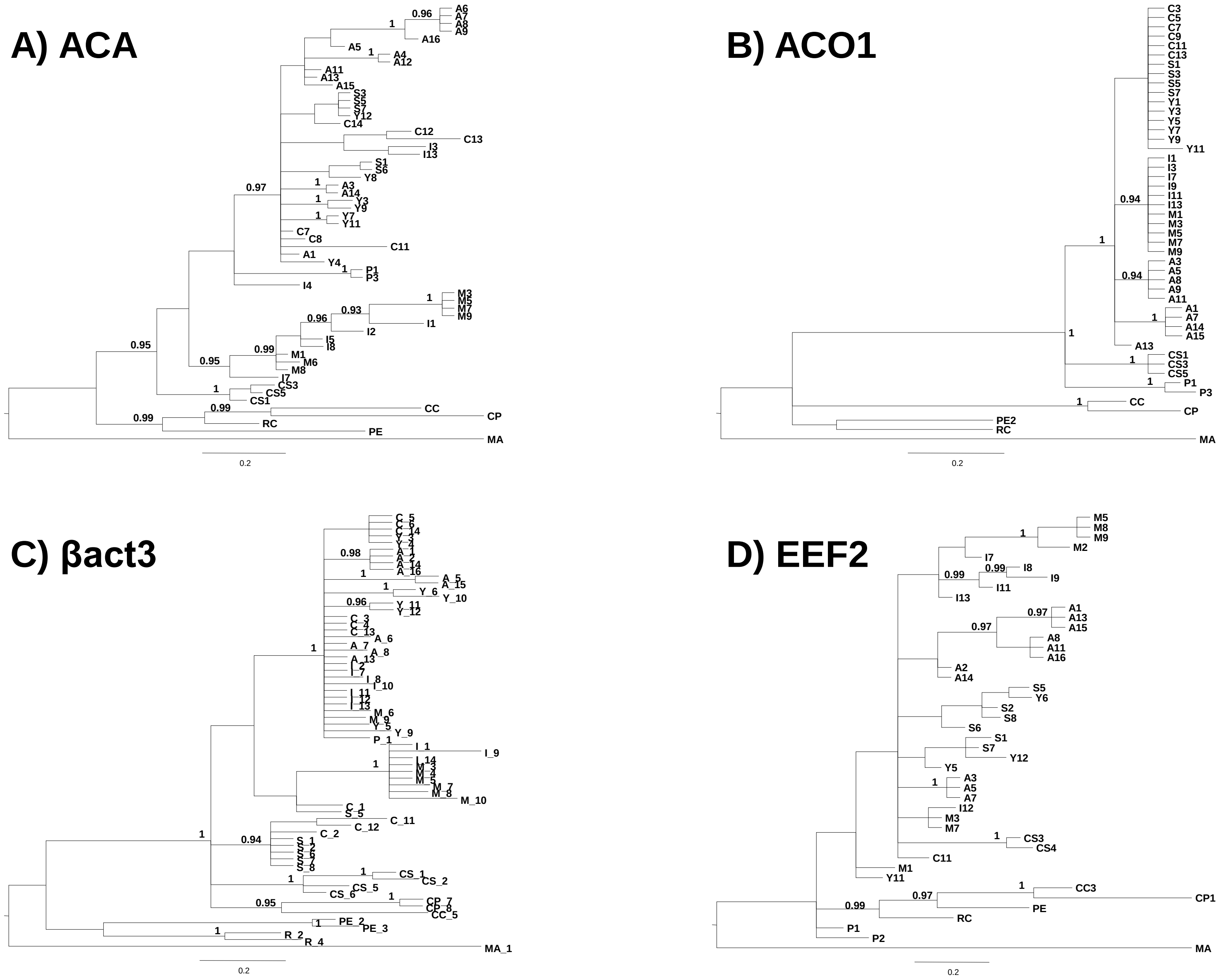
A) ACA
A6
A7
A8
A9
A16
A5
A4
A12
A11
A13
A15
S3
S5
S7
Y12
C14
C12
C13
I3
I13
S1
S6
Y8
A3
A14
Y3
Y9
Y7
Y11
C7
C8
C11
A1
Y4
P1
P3
I4
M3
M5
M7
M9
I1
I2
I5
I8
M1
M6
M8
I7
CS3
CS5
CS1
CC
CP
RC
PE
MA
0.2
B) ACO1
C3
C5
C7
C9
C11
C13
S1
S3
S5
S7
Y1
Y3
Y5
Y7
Y9
Y11
I1
I3
I7
I9
I11
I13
M1
M3
M5
M7
M9
A3
A5
A8
A9
A11
A1
A7
A14
A15
A13
CS1
CS3
CS5
P1
P3
CC
CP
PE2
RC
MA
0.2
C) βact3
A_5
A_15
Y_6
Y_10
Y_11
Y_12
C_3
C_4
C_13
A_6
A_7
A_8
A_13
I_8
I_10
M_6
M_9
Y_5
Y_9
P_1
I_1
I_9
I_14
M_3
M_7
M_8
M_10
C_1
S_5
C_11
C_12
C_2
S_8
CS_1
CS_2
CS_5
CS_6
CP_7
CC_5
PE_2
PE_3
R_2
R_4
MA_1
0.2
D) EEF2
M5
M8
M9
M2
I7
I8
I9
I11
I13
A1
A13
A15
A8
A11
A16
A2
A14
S5
Y6
S2
S8
S6
S1
S7
Y12
Y5
A3
A5
A7
I12
M3
M7
CS3
CS4
C11
M1
Y11
CC3
CP1
PE
RC
P1
P2
MA
0.2

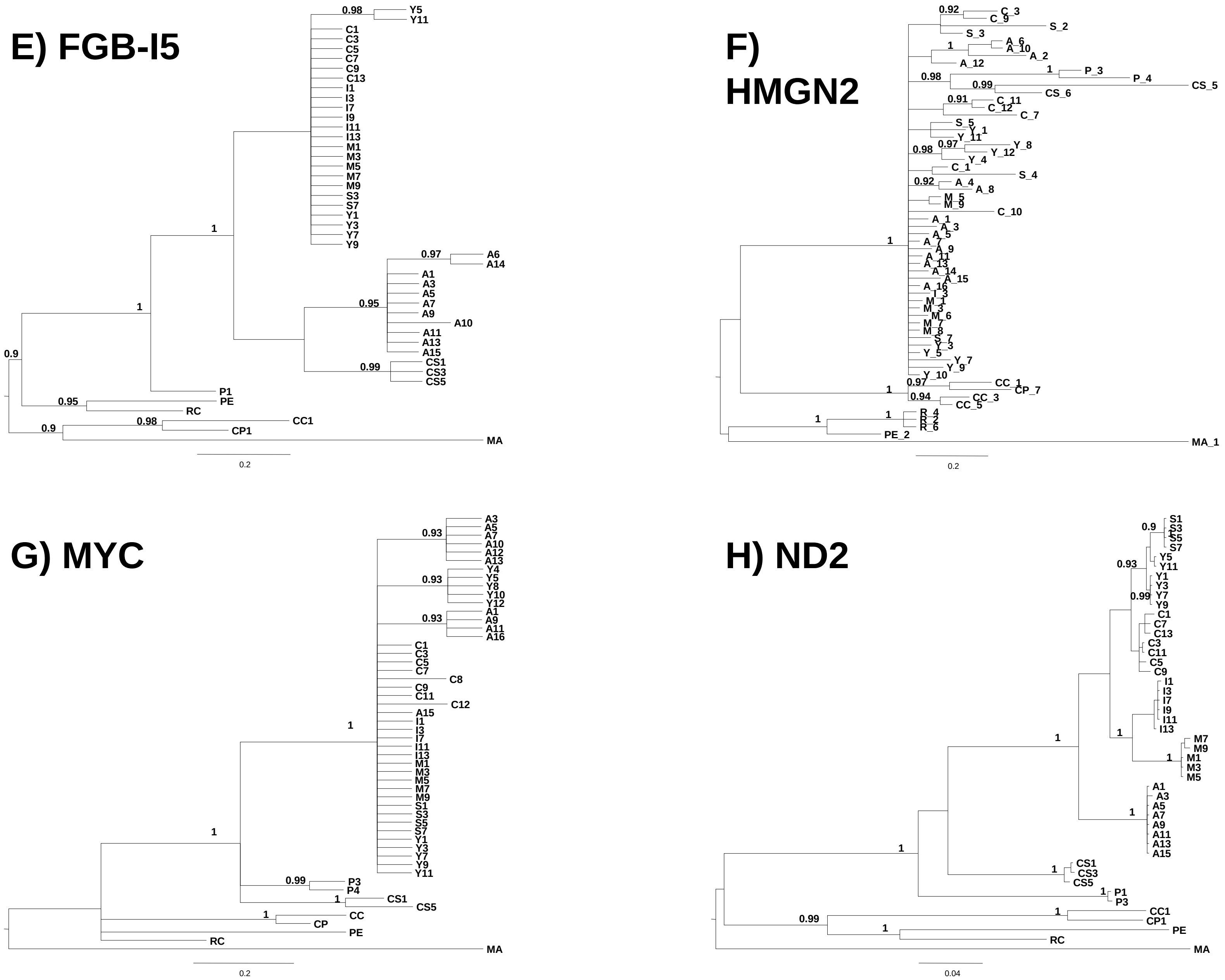
E) FGB-I5
Y5
Y11
C1
C3
C5
C7
C9
C13
I1
I3
I7
I9
I11
I13
M1
M3
M5
M7
M9
S3
S7
Y1
Y3
Y7
Y9
A6
A14
A1
A3
A5
A7
A9
A10
A11
A13
A15
CS1
CS3
CS5
P1
PE
RC
CC1
CP1
MA
0.98
1
1
0.97
0.95
0.99
0.9
0.95
0.98
0.9
0.2
F) HMGN2
C_3
C_9
S_2
S_3
A_6
A_10
A_2
A_12
P_3
P_4
CS_5
CS_6
C_11
C_12
C_7
S_5
Y_1
Y_11
Y_8
Y_12
Y_4
C_1
S_4
A_4
A_8
M_5
M_9
C_10
A_1
A_3
A_5
A_7
A_9
A_11
A_13
A_14
A_15
A_16
I_3
M_1
M_3
M_6
M_7
M_8
S_7
Y_3
Y_5
Y_7
Y_9
Y_10
CC_1
CP_7
CC_3
CC_5
R_4
R_2
R_6
PE_2
MA_1
0.92
1
1
0.98
0.99
0.91
0.97
0.98
0.92
1
0.97
1
0.94
1
1
0.2
G) MYC
A3
A5
A7
A10
A12
A13
Y4
Y5
Y8
Y10
Y12
A1
A9
A11
A16
C1
C3
C5
C7
C8
C9
C11
C12
A15
I1
I3
I7
I11
I13
M1
M3
M5
M7
M9
S1
S3
S5
S7
Y1
Y3
Y7
Y9
Y11
P3
P4
CS1
CS5
CC
CP
PE
RC
MA
0.93
0.93
0.93
1
1
0.99
1
1
0.2
H) ND2
S1
S3
S5
S7
Y5
Y11
Y1
Y3
Y7
Y9
C1
C7
C13
C3
C11
C5
C9
I1
I3
I7
I9
I11
I13
M7
M9
M1
M3
M5
A1
A3
A5
A7
A9
A11
A13
A15
CS1
CS3
CS5
P1
P3
CC1
CP1
PE
RC
MA
0.9
1
0.93
0.99
1
1
1
1
1
1
1
1
0.99
1
0.04

I) ODC

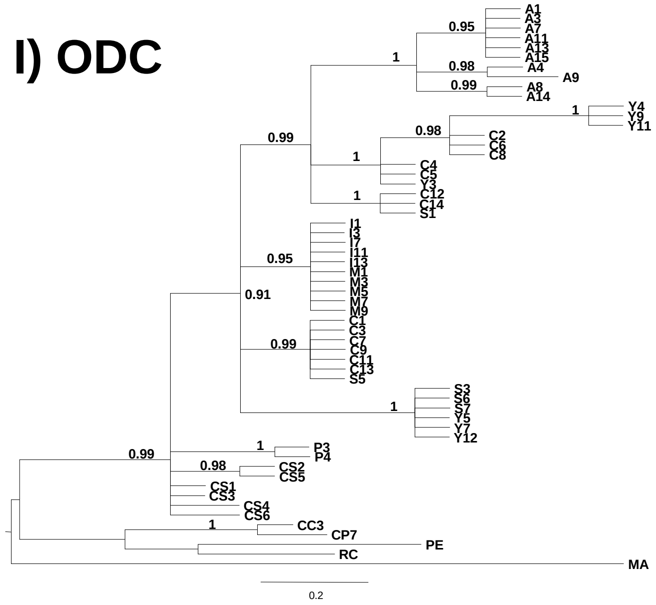

A1
A3
A7
A11
A13
A15
0.95
1
0.98
A4
A9
0.99
A8
A14
1
Y4
Y9
Y11
0.98
C2
C6
C8
0.99
1
C4
C5
Y3
1
C12
C14
S1
I1
I3
I7
I11
I13
M1
M3
M5
M7
M9
0.95
0.91
0.99
C1
C3
C7
C9
C11
C13
S5
1
S3
S6
S7
Y5
Y7
Y12
0.99
1
P3
P4
0.98
CS2
CS5
CS1
CS3
CS4
CS6
1
CC3
CP7
PE
RC
MA
0.2

J) RHO-I1

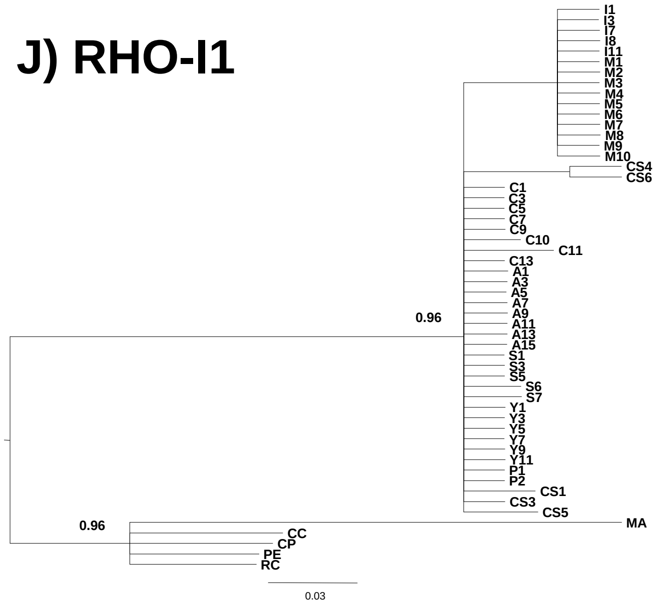

I1
I3
I7
I8
I11
M1
M2
M3
M4
M5
M6
M7
M8
M9
M10
CS4
CS6
C1
C3
C5
C7
C9
C10
C11
C13
A1
A3
A5
A7
A9
A11
A13
A15
S1
S3
S5
S6
S7
Y1
Y3
Y5
Y7
Y9
Y11
P1
P2
CS1
CS3
CS5
0.96
0.96
MA
CC
CP
PE
RC
0.03
