# Supplementary figures and images for: Examining the Role of Effective Population Size on Mitochondrial and Multilocus Divergence Time Discordance in a Songbird
Source: PLoS One. 2013 Feb 15;8(2):e55161. doi: 10.1371/journal.pone.0055161 (PMC3574149; doi:10.1371/journal.pone.0055161)

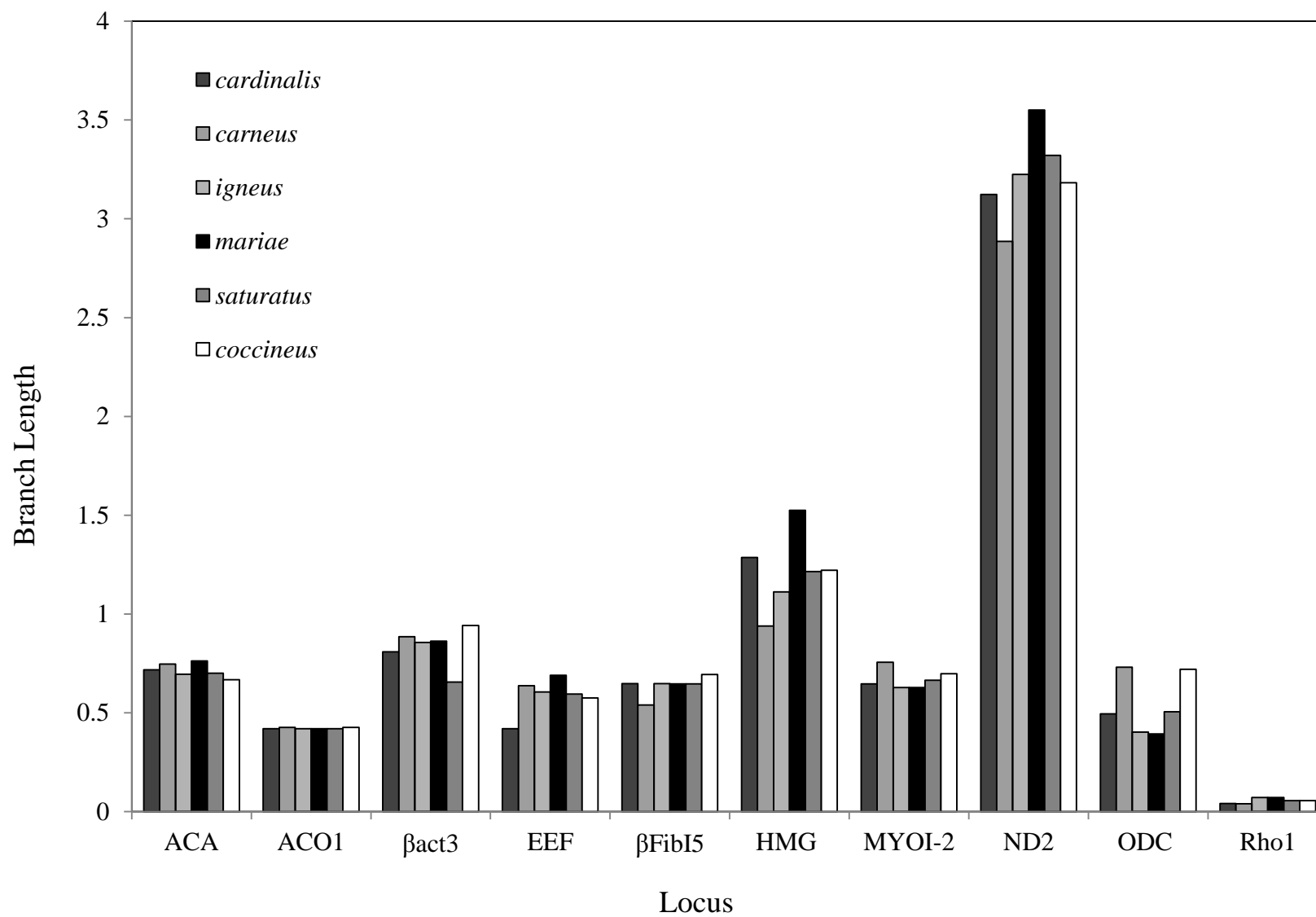

Supplement: Figure S2 — Cardinalis cardinalis mean branch lengths. Branch lengths were extracted from gene trees constructed using MrBayes. The branch length was the distance from each lineage to a common outgroup, Cardinalis sinuatus. Mean branch lengths were generated by averaging across all the individuals within a lineage. (PDF) [file pone.0055161.s002.pdf]
