# Supplementary material for: Examining the Role of Effective Population Size on Mitochondrial and Multilocus Divergence Time Discordance in a Songbird
Source: PLoS One. 2013 Feb 15;8(2):e55161. doi: 10.1371/journal.pone.0055161 (PMC3574149; doi:10.1371/journal.pone.0055161)
Supplement: Table S1 — Molecular clock likelihood ratio tests. Shown are locus name, sequence model, and likelihood scores for clock constrained and unconstrained for each gene tree. Gene trees that are inconsistent with the molecular clock are shown in bold. (PDF) [file pone.0055161.s003.pdf]

Table S1 Molecular clock likelihood ratio tests

**locus**	**substitution model**	**unconstrained lk**	**clock constrained lk**	**df**	**2Δ**	**0.05**
ACA	JC	-2328.19	-2378.24	55	**100.11**	73.31
ACO1	JC	-1331.75	-1336.06	70	8.62	90.53
Bact3	JC	-870.59	-900.72	52	60.25	69.83
EEF2	JC	-1402.89	-1433.58	44	61.38	61.66
FGB-I5	JC	-816.64	-824.57	66	15.86	85.97
HMGN2	JC	-1765.68	-1829.29	45	**127.21**	61.66
MYC	JC	-1047.12	-1056.98	66	19.73	85.97
ND2	JC	-2341.79	-2363.28	35	42.98	49.80
ODC	JC	-1046.05	-1059.34	66	26.59	85.97
RHO-I1	JC	-422.90	-429.94	68	14.08	88.25
ACA	GTR + I + G	-2354.13	-2442.20	55	**176.14**	73.31
ACO1	HKY	-1483.47	-1605.84	70	**244.73**	90.53
Bact3	HKY + I + G	-899.20	-937.90	52	**77.41**	69.83
EEF2	HKY + I + G	-1422.00	-1481.02	44	**118.05**	61.66
FGB-I5	HKY	-855.89	-908.62	66	**105.46**	85.97
HMGN2	GTR + I + G	-1832.29	-1961.58	45	**258.57**	61.66
MYC	K80	-1108.34	-1152.74	66	**88.79**	85.97
ND2	GTR + G	-2653.56	-2817.04	35	**326.96**	49.80
ODC	HKY	-1065.78	-1103.44	66	75.32	85.97
RHO-I1	HKY + I	-451.03	-486.02	68	69.99	88.25
