# Supplementary material for: Examining the Role of Effective Population Size on Mitochondrial and Multilocus Divergence Time Discordance in a Songbird
Source: PLoS One. 2013 Feb 15;8(2):e55161. doi: 10.1371/journal.pone.0055161 (PMC3574149; doi:10.1371/journal.pone.0055161)
Supplement: Table S2 — Speciation time estimated from mtDNA and multilocus data. (PDF) [file pone.0055161.s004.pdf]

Table S2. Speciation time estimates from mtDNA and multilocus data

	node	mean (mya)	95 % CI		mean (mya)	95% CI
**1A**	A	2.35	(1.81 - 2.93)	**1B**	2.03	(1.52 - 2.54)
	B	1.75	(1.27 - 2.24)		1.23	(0.85 - 1.63)
	C	0.70	(0.33 - 1.05)		0.40	(0.23 - 0.62)
	D	0.18	(0.02 - 0.38)		0.07	(0.01 - 0.20)
	E	1.15	(0.58 - 1.58)		0.19	(0.07 - 0.36)
**2A**	A	1.82	(1.38 – 2.28)	**2B**	1.85	(1.02 - 2.85)
	B	1.52	(1.09 – 1.96)		1.47	(0.76 - 2.27)
	C	0.44	(0.26 – 0.66)		0.46	(0.20 - 0.78)
	D	0.22	(0.09 - 0.35)		0.23	(0.07 - 0.42)
	E	0.26	(0.09 – 0.46)		0.26	(0.07 – 0.48)
**3A**	A	1.78	(1.26 - 2.36)	**3B**	1.74	(1.18 - 2.34)
	B	1.43	(0.97 - 1.87)		1.37	(0.94 - 1.79)
	C	0.41	(0.24 - 0.59)		0.41	(0.24 - 0.59)
	D	0.17	(0.06 - 0.29)		0.17	(0.07 - 0.28)
	E	0.23	(0.09 - 0.40)		0.23	(0.07 - 0.39)
**4A**	A	2.41	(1.31 - 3.77)	**4B**	2.56	(1.38 - 4.01)
	B	1.87	(1.05 - 2.80)		1.89	(1.10 - 2.85)
	C	1.00	(0.46 - 1.66)		1.02	(0.45 - 1.69)
	D	0.33	(0.12 - 0.59)		0.32	(0.12 - 0.56)
	E	1.07	(0.45 - 1.76)		1.08	(0.46 - 1.76)
**5A**	A	1.88	(1.08 - 2.79)	**5B**	1.93	(1.10 - 2.89)
	B	1.54	(0.86 - 2.31)		1.55	(0.86 - 2.33)
	C	0.48	(0.23 - 0.78)		0.48	(0.22 - 0.78)
	D	0.21	(0.06 - 0.37)		0.20	(0.07 - 0.34)
	E	0.28	(0.11 - 0.49)		0.28	(0.10 - 0.49)

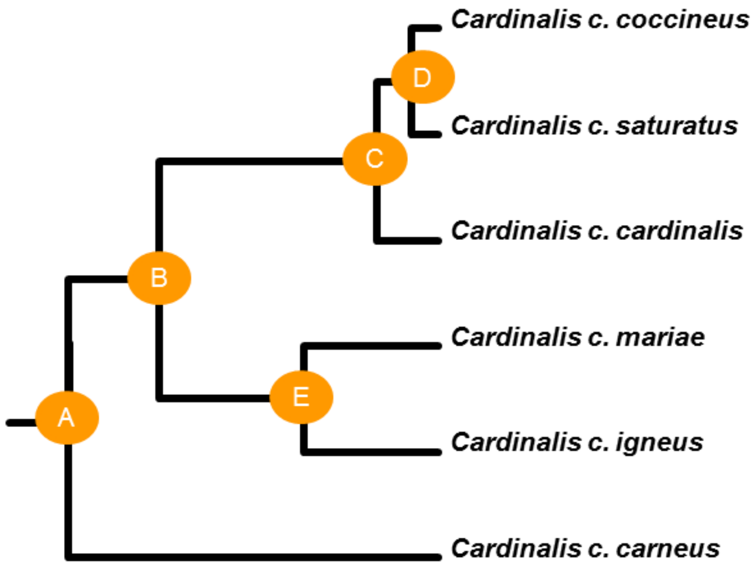

The node column corresponds to a divergence in the species tree shown in the figure above. Numbers (1-5) and letters (A & B) correspond to the following speciation time analyses: 1 - MCMCcoal estimate: A) mtDNA only B) multilocus (mtDNA and introns), D* = speciation times estimate for Node D using an Early Pleistocene prior (1, 40) D* A) 0.26 (0.05-0.49)   B) D* 0.10 (0.01-0.24). The inclusion of this prior distribution did not change the estimates at the other nodes; 2 - *BEAST only introns: A) strict clock B) relaxed clock; 3 - *BEAST strict clock for mtDNA using a strict clock for introns: A) ND2 unpartitioned B) ND2 partitioned; 4 - *BEAST mtDNA only using a relaxed clock: A) ND2 unpartitioned B) ND2 partitioned ; and 5 - *BEAST speciation times using a relaxed clock for mtDNA and introns: A) ND2 unpartitioned B) ND2 partitioned.
