# Supplementary material for: Examining the Role of Effective Population Size on Mitochondrial and Multilocus Divergence Time Discordance in a Songbird
Source: PLoS One. 2013 Feb 15;8(2):e55161. doi: 10.1371/journal.pone.0055161 (PMC3574149; doi:10.1371/journal.pone.0055161)
Supplement: Table S3 — Substitution rates and branch lengths extracted from the mtDNA gene tree. (PDF) [file pone.0055161.s005.pdf]

Table S3 Substitution rates and branch lengths.

ND2 haplotype	dN	dS	dN/dS	BL
A1	0.004587	0.108657	0.042217	2.886574
A3	0.004587	0.135714	0.033800	2.886070
A5	0.004587	0.103458	0.044338	2.886005
A7	0.004587	0.128692	0.035645	2.886005
C1	0.001524	0.026407	0.057727	3.216227
C3	0.000000	0.013337	0.000000	3.020157
C5	0.000000	0.035139	0.000000	3.103538
C7	0.000000	0.021523	0.000000	3.151217
I1	0.001524	0.024199	0.062994	3.202938
I3	0.001524	0.032541	0.046845	3.203484
I9	0.001524	0.024199	0.062994	3.202552
I7	0.001524	0.035373	0.043095	3.203127
M1	0.009231	0.051334	0.179823	3.549841
M3	0.009231	0.045660	0.202170	3.549306
M5	0.009231	0.054315	0.169952	3.548721
M7	0.009231	0.051334	0.179823	3.555508
S1	0.001524	0.026492	0.057541	3.321141
S3	0.000000	0.015564	0.000000	3.321154
S5	0.000000	0.015598	0.000000	3.321952
S7	0.000000	0.015564	0.000000	3.321677
Y1	0.001203	0.032736	0.036754	3.157226
Y3	0.001203	0.016003	0.075184	3.158116
Y5	0.000000	0.005301	0.000000	3.208751
Y7	0.001203	0.016003	0.075184	3.208537

Shown are substitution rates and branch lengths for each *Cardinalis cardinalis* haplotype. Letters and numbers correspond to haplotype codes for the different lienages: *cardinalis* (C); *carneus* (A); *igneus* (I); *mariae* (M); *saturatus* (S); *coccineus* (Y). Nonsynonymous amino acid substitution rate dN; synonymous amino-acid substitution rate (dS); ND2 branch length estimated from a common outgroup (BL)
